# Comparison of pulmonary and aortic root and cusp dimensions in normal adults using computed tomography: potential implications for Ross procedure planning

**DOI:** 10.1093/icvts/ivae206

**Published:** 2024-12-09

**Authors:** Matija Jelenc, Blaž Jelenc, Sara Habjan, Karen B Abeln, Peter Fries, Hector I Michelena, Hans Joachim Schäfers

**Affiliations:** Department of Cardiovascular Surgery, University Medical Centre Ljubljana, Ljubljana, Slovenia; Faculty of Mathematics and Physics, University of Ljubljana, Ljubljana, Slovenia; Department of Cardiovascular Surgery, University Medical Centre Ljubljana, Ljubljana, Slovenia; Department of Thoracic and Cardiovascular Surgery, Saarland University Medical Centre, Homburg/Saar, Germany; Clinic for Diagnostic and Interventional Radiology, Saarland University Medical Centre, Homburg/Saar, Germany; Department of Cardiovascular Medicine, Mayo Clinic, Rochester, MN, USA; Department of Cardiac Surgery, Hospital Universitario Quironsalud, Madrid, Spain; Department of Thoracic and Cardiovascular Surgery, Westpfalz Klinikum, Kaiserslautern, Germany; Saarland University, Saarbrücken, Germany

**Keywords:** Computed tomography angiography, Pulmonary valve anatomy, Ross procedure, Aortic valve anatomy, Cardiac cycle

## Abstract

**OBJECTIVES:**

The Ross procedure is currently receiving renewed interest. Its function and durability depend on preservation of pulmonary valve anatomy; limited data exist on normal pulmonary valve geometry. The objective was to compare aortic and pulmonary root and cusp dimensions in adults with normal tricuspid aortic and pulmonary valves.

**METHODS:**

We reviewed 507 coronary computed tomography studies, selecting those with adequate visibility of both pulmonary and aortic roots for further analysis. Diastolic aortic and pulmonary root and cusp dimensions were measured. Root dimensions at different phases of the cardiac cycle were measured in 3 patients.

**RESULTS:**

We analysed studies of 50 patients with the mean age of 54 years [standard deviation (SD): 16]. In end-diastole, pulmonary root had a smaller sinutubular to basal ring ratio than the aortic root [0.82 (SD: 0.09) vs 1.14 (SD: 0.12), *P* < 0.001]. Aortic and pulmonary cusps had similar dimensions; however, pulmonary cusp effective height was lower [5.9 mm (SD: 1.6) vs 8.4 mm (SD: 1.2), *P* < 0.001]. Pulmonary basal ring perimeter was largest at end-diastole and smallest at end-systole, with the relative difference of 23.5% (SD: 2.7).

**CONCLUSIONS:**

The pulmonary root has a similar cusp size compared to the aortic root, but a different shape, resulting in a lower pulmonary cusp effective height. The perimeter of the pulmonary basal ring changes during the cardiac cycle. These findings suggest that stabilizing the autograft to normal aortic, rather than pulmonary, root dimensions should result in normal autograft cusp configuration. Computed tomography angiography could become a tool for personalized planning of the Ross procedure.

## Graphical abstract

**Figure:**
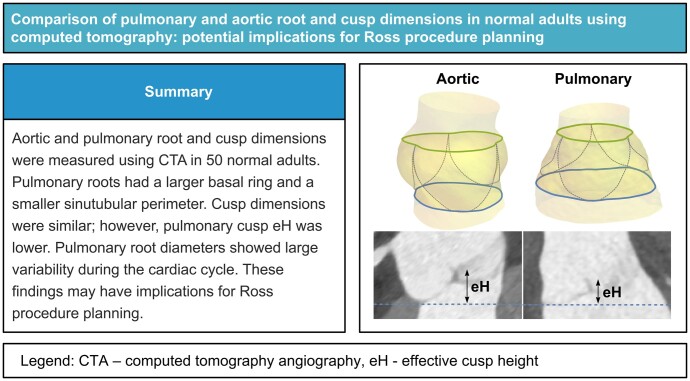

## INTRODUCTION

The pulmonary artery autograft is increasingly used to replace the diseased aortic valve in the setting of the Ross procedure in young adults. Recent studies have shown that it is associated with excellent haemodynamics, better quality of life, better long-term survival and higher freedom from valve-related complications compared to prosthetic aortic valve replacement [1–5].

An important prerequisite for autograft durability is the preservation of normal autograft geometry during the procedure. Later, exposure to systemic arterial pressure may lead to autograft dilatation and valve regurgitation [6]. While the relationship between cusp and root dimensions in the aortic valve has been characterized [7, 8], little is known about normal pulmonary root and valve dimensions.

The most accurate measurements of the pulmonary root and cusps come from older studies on cadavers and thus may not reflect the *in vivo* conditions [9–11]. Compared to computed tomography, 2D transthoracic and transoesophageal echocardiography data have limited value due to dependence on gain settings and availability of ultrasound windows, as well as the inability to precisely measure the dimensions of the pulmonary basal ring (BR) and sinutubular junction (STJ) in orthogonal views [12]. Adequate visualization of the pulmonary root can be achieved with 3D echocardiography in only 45–75% of patients [12].

Anatomical differences compared to the aortic valve also relate to the BR. In the aortic valve, it is ∼42% muscular and 58% fibrous [13], while in the pulmonary valve, the BR is entirely supported by muscular sub-pulmonary infundibulum [14]. An important consideration is also the difficulty of clearly imaging the thin pulmonary valve cusps with any of the currently available imaging modalities.

In the past years, computed tomography angiography (CTA) imaging has become increasingly used for the preoperative assessment of the aortic valve, as it allows for precise determination of *in vivo* root and cusp dimensions [8, 15, 16]. It is yet unknown whether it can also be used to measure details of the pulmonary valve and root. Such details, determined *in vivo*, could provide the basis for planning of the Ross procedure.

The objective of the current study was to visualize and measure aortic and pulmonary root and cusp dimensions in adults with normal tricuspid aortic and pulmonary valves, using CTA. Pairwise comparison of aortic and pulmonary root and cusp dimensions was then performed, using the measurements of the aortic valve and root as individual controls.

## PATIENTS AND METHODS

### Study design

We retrospectively analysed electrocardiogram-gated and anonymized coronary CTA studies of adult patients obtained from our hospital’s picture archiving and communication system. The CTAs were performed to rule out coronary disease or anomalies between December 2017 and February 2024. CTA datasets included gender, age, height and weight of the patient with all other metadata being anonymized. Initially, the CTAs were selected randomly; however, as the number of patients grew, we tried to keep the male to female ratio at approximately 1:1, thus adding more female or male patients as needed.

### Ethics

The study was approved by the National Medical Ethics Committee [NMEC; Komisija Republike Slovenije za medicinsko etiko 0120-133/2021/3 (14 May 2021), 0120-312/2022/3 (5 September 2022) and 0120-25/2024-2711-6 (24 April 2024)]. Written patient informed consent was waived as this was a non-interventional study with retrospective data acquisition and analysis.

### Inclusion and exclusion criteria

Only patients with normally appearing tricuspid aortic and pulmonary valves and normal aortic root size, defined as the diameter of sinuses of Valsalva <40 mm (largest sinus-to-sinus distance), were included in the study. CTAs were excluded from further analysis in the presence of 1 or more exclusion criteria. Exclusion criteria were as follows: poor pulmonary cusp visibility, poor contrast in the pulmonary root, cranial part of the pulmonary root missing on the CTA, aortic cusp calcifications, motion artefacts, root diameter larger than 40 mm and bicuspid aortic valve. Adequate contrast in the pulmonary artery and adequate visibility of pulmonary valve cusps allowed simultaneous aortic and pulmonary valve analysis.

### Valve analysis

**Figure 1:**
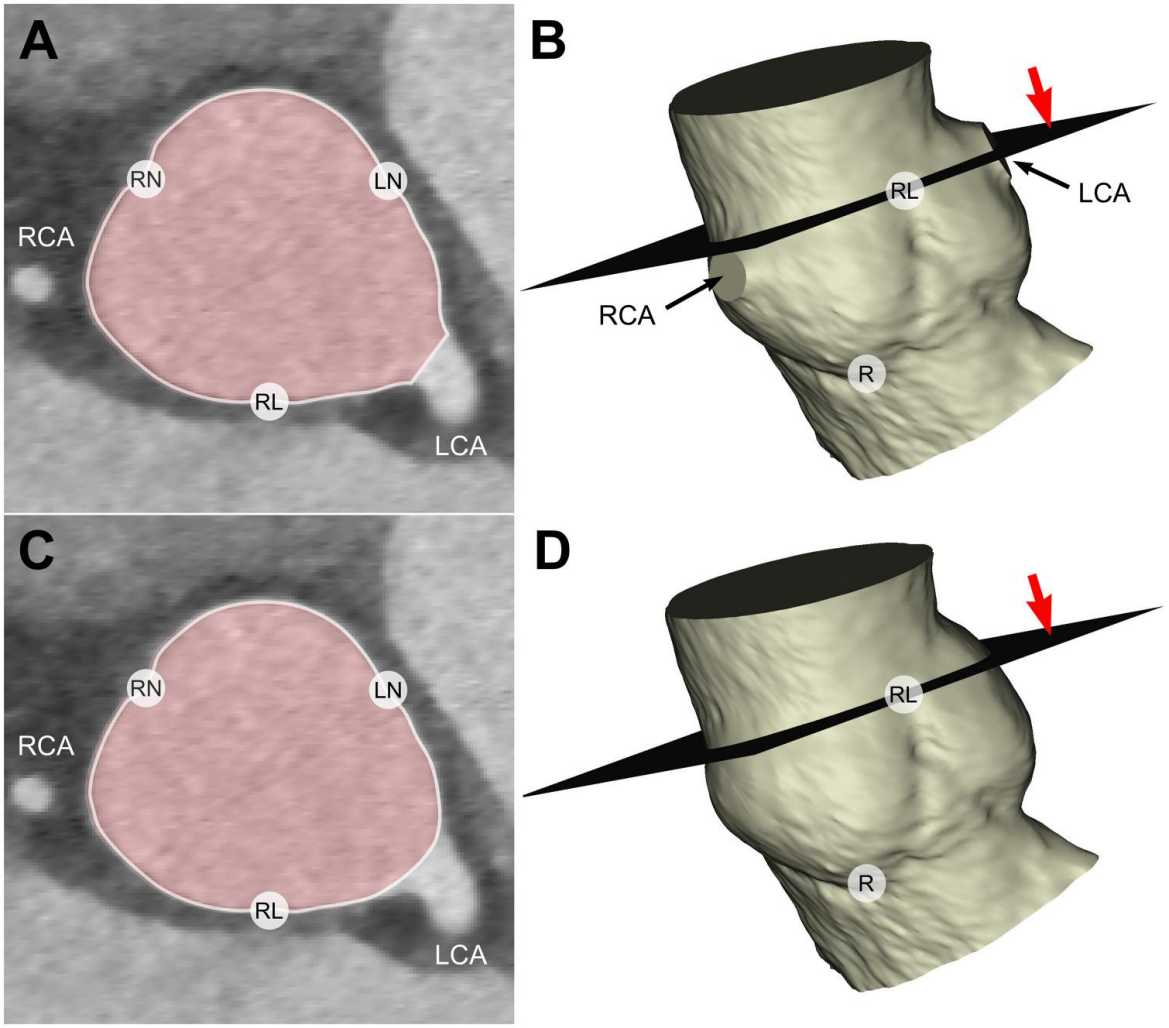
Aortic commissural perimeter measurement in patients with commissural plane transecting 1 or both coronary ostia. (**A**) View of the aortic root at the commissural plane. (**B**) 3D model of the segmented root, with commissural plane marked with red arrow. Coronary arteries are cut flush at the ostia. (**C**) Same aortic root with the 3D model smoothed at the coronary ostia. (**D**) 3D model with sinuses smoothed, appearing as if there were no coronary ostia. LCA: left coronary artery; LN: left noncoronary commissure; R: nadir of the right sinus; RCA: right coronary artery; RL: right left commissure; RN: right noncoronary commissure.

The CTAs were imported into Mimics Innovation Suite v. 21.0 (Materialise, Leuven, Belgium) where aortic and pulmonary roots were segmented. On each CTA, we marked the nadirs of the 3 sinuses and the 3 commissures for both the aortic and pulmonary valves. The spline tool was used to trace the aortic and pulmonary cusp insertions and geometric heights as well as aortic and pulmonary BR at level of nadirs of the 3 sinuses. Commissural perimeter of the root was measured at the plane defined by the 3 commissures. In aortic roots, 1 or both coronary ostia frequently branched off the root at the commissural plane level. To avoid the interference with commissural perimeter measurement, the 3D aortic root models were smoothed at the coronary ostia using Blender software (Blender Foundation, opensource) (Fig. 1). STJ perimeter was measured at the aortic or pulmonary narrowing above the sinuses, which was a few millimetres higher than the plane of the 3 commissures.

The points and splines, defined by 3D coordinates and the .stl files of the segmented roots, were then imported into Mathematica v12.0 (Wolfram Research, Champaign, IL, USA) where a dedicated code was used to reconstruct the aortic and pulmonary root in 3D space from the measured points and splines and calculate different measurements for each patient. The BR spline was the basis for measurements of BR perimeter, as well as maximal and minimal BR diameter. Maximal and minimal diameters were defined as the longest and the shortest diameters passing through the centroid of the BR, respectively. The degree of ellipticity was estimated by calculating the ratio between minimal and maximal BR diameter. Mean BR, STJ and commissural diameters were calculated as respective perimeters divided by pi.

Cusp effective height was calculated as the distance between the BR plane, defined by the 3 nadirs of the sinuses, and the tip of the geometric height spline. The free margin length of a cusp was estimated by adding the distances from the 2 corresponding commissures to the tip of the geometric height spline. The entire process of valve analysis was shown in a video previously [17].

Additionally, 3 patients had usable CTA images at 30, 40, 50, 60, 70 and 80% of the R–R interval, allowing measurement of changes in pulmonary root and aortic root dimensions during the cardiac cycle.

### Statistical methods

Statistical data analysis was performed using JASP v 0.14.1 (University of Amsterdam, Netherlands). Normal distribution of variables was assessed using the Shapiro–Wilk test. Continuous variables are reported as mean and standard deviation (SD) if normally distributed and as median and interquartile range otherwise. Differences between the patients were analysed using Student’s *t*-test. Differences between the aortic and pulmonary root were analysed using paired samples *t*-test, Wilcoxon signed-rank test and repeated measures ANOVA with *post-hoc* test with Bonferroni correction. *P*-value of <0.05 was considered statistically significant.

## RESULTS

**Figure 2:**
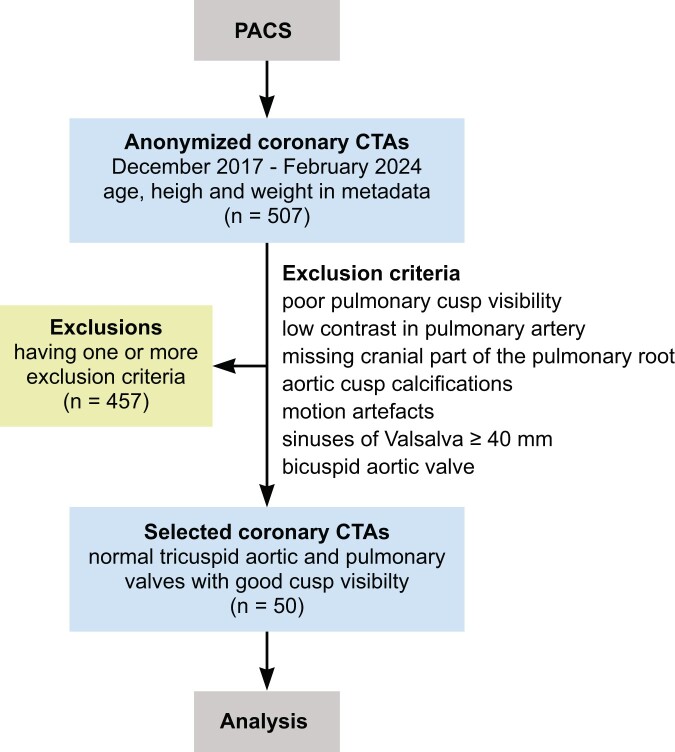
Study flowchart. CTA: computed tomography angiography; PACS: hospital’s picture archiving and communication system.

We reviewed 507 coronary CTAs. Four hundred fifty-seven CTAs were excluded due to 1 or more exclusion criteria leaving 50 CTAs for detailed analysis (Fig. 2). Incidence of exclusion criteria was as follows: poor cusp visibility in 388 (84.9%), low contrast in the pulmonary artery in 338 (74.0%), missing cranial part of the pulmonary root in 178 (38.9%), aortic cusp calcifications in 27 (5.9%), motion artefacts in 22 (4.8%), aortic root size more than 40 mm in 13 (2.8%) and bicuspid aortic valve in 9 (2.0%). The number of exclusion criteria per excluded CTA was as follows: 1 in 77 (16.8%), 2 in 255 (55.8%), 3 in 105 (23.0%) and 4 in 18 (3.9%).

Detailed analysis was performed on CTAs of the 50 patients who met the inclusion criteria. All valve analyses were performed in end-diastolic phase of the cardiac cycle with R–R interval at a median of 75 (interquartile range: 4)% of the cardiac cycle. Median heart rate at acquisition was 65 (interquartile range: 8) beats per minute. Slice thickness was 0.6 mm. CTAs were acquired using different systems from 1 vendor (41 Somatom Force, 9 Somatom Drive, both Siemens Healthineers, Erlangen, Germany).

Mean patient age was 54 years (SD: 16, range: 19–76), 27 patients (54%) were male, mean height and weight were 172 cm (SD: 10, range: 150–191) and 77 kg (SD: 13, range: 55–106), respectively. Mean body surface area (BSA) was 1.91 m^2^ (SD: 0.21, range: 1.59–2.35). Women were older [60 (SD: 13) vs 50 (SD: 17) years, *P* = 0.029, Student’s *t*-test] and had smaller BSA [1.75 (SD: 0.13) vs 2.06 (SD: 0.15) m^2^, *P* < 0.001, Student’s *t*-test].

**Figure 3:**
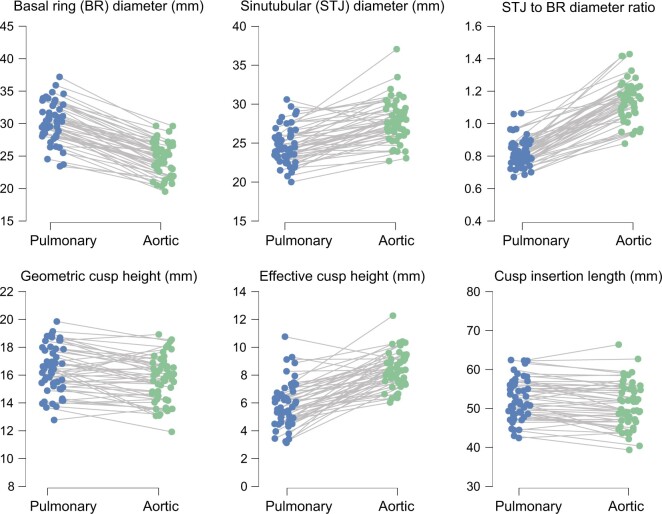
Case-by-case differences in selected root and cusp measurements.

The diastolic shape of the pulmonary root was different from the aortic root. BR diameter was larger in pulmonary roots, whereas the STJ and commissural diameters were smaller (Table 1; Fig. 3; Supplementary Material, Fig. S1). Consequently, the STJ to BR ratio was larger in aortic roots [1.14 (SD: 0.12) vs 0.82 (SD: 0.09), *P* < 0.001] with a mean difference of 0.32 (95% CI 0.29, 0.35). Also, the BR ellipticity ratio was smaller in pulmonary roots (signifying a more elliptical and less circular shape).

**Table 1:** Comparison of pulmonary and aortic root and cusp dimensions

	Aortic root (*n* = 50)	Pulmonary root (*n* = 50)	*P*-value^a^
	Mean (SD)	Mean (SD)	
Basal ring diameter (mm)	24.6 (2.7)	30.2 (3.0)	<0.001
Basal ring ellipticity ratio^b^	0.77 (0.05)	0.64 (0.09)	<0.001
Basal ring area (mm^2^)	451 (95)	645 (131)	<0.001
Sinutubular diameter (mm)	27.8 (2.7)	24.6 (2.4)	<0.001
Sinutubular area (mm^2^)	599 (123)	473 (93)	<0.001
Sinutubular to basal ring ratio	1.14 (0.12)	0.82 (0.09)	<0.001
Commissural diameter (mm)	31.3 (3.3)	26.7 (2.9)	<0.001
Commissural area (mm^2^)	741 (151)	540 (118)	<0.001
Mean cusp insertion (mm)	51.0 (5.8)	52.6 (5.2)	<0.001
Mean commissural height (mm)	19.4 (2.3)	19.4 (2.0)	0.981
Mean intercommissural distance (mm)	23.4 (2.2)	20.4 (2.0)	<0.001
Mean geometric height (mm)	15.6 (1.7)	16.4 (1.7)	<0.001
Mean effective height (mm)	8.4 (1.2)	5.9 (1.6)	<0.001
Mean estimated free margin length (mm)	33.5 (3.9)	34.2 (4.0)	0.069
Root volume (cm^3^)	12.3 (3.6)	13.9 (4.3)	<0.001

a Paired samples *t*-test.

b Ellipticity ratio is defined as minimal/maximal diameter.

The cusp measurements, apart from mean effective cusp height (eH), were similar in aortic and pulmonary valves. Estimated free margin length and commissural height were identical. Cusp insertion length and geometric cusp height were larger in pulmonary roots (*P* < 0.001); however, the difference was small (0.8 mm in geometric cusp height and 1.6 mm in cusp insertion length) (Table 1; Fig. 3; Supplementary Material, Fig. S1).

**Figure 4:**
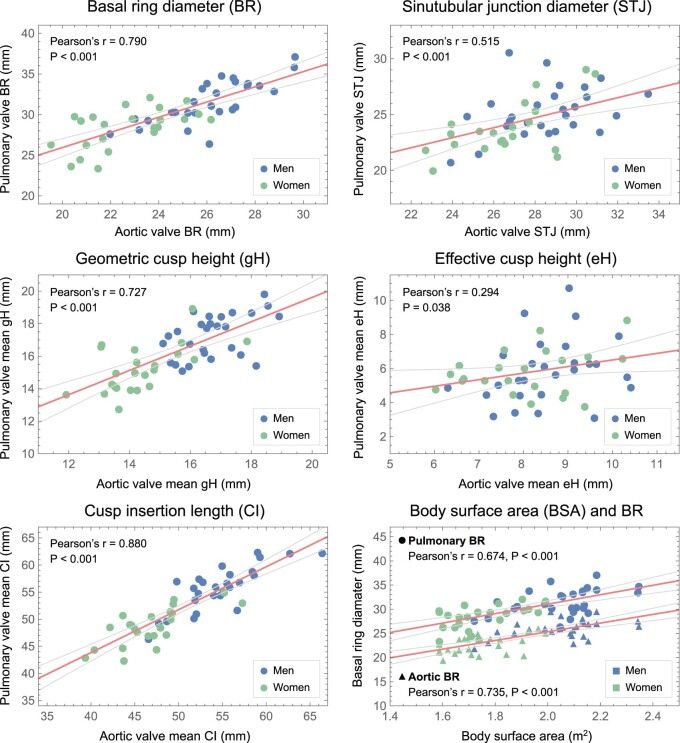
Correlations between corresponding aortic and pulmonary measurements for basal ring (BR) diameter, sinutubular junction diameter (STJ), mean cusp geometric height (gH), mean cusp effective height (eH) and mean cusp insertion length (CI). Note the strong correlation for BR, gH and CI, moderate for STJ and weak for eH. Both pulmonary and aortic BR show positive correlation with body surface area (BSA).

Pulmonary root and cusp dimensions showed strong correlation with aortic root and cusp dimensions, except for eH, which showed only weak correlation (Fig. 4 and Supplementary Material, Table S1). Root and cusp dimensions correlated positively with BSA.

### Pulmonary root during cardiac cycle

Three patients had usable CTA images of the aortic and pulmonary root at 30–80% of the R–R interval. Two were female aged 29 and 53 years and 1 was a 72-year-old male. Their BSAs were 1.73, 1.81 and 2.14 m^2^, respectively.

**Figure 5:**
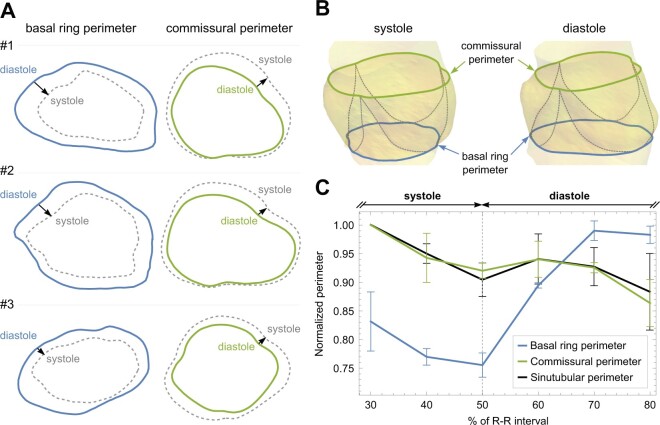
Pulmonary root dimensions during cardiac cycle. (**A**) Three patients with maximal and minimal pulmonary basal ring and commissural perimeters in end-systole and end-diastole. (**B**) 3D volume renderings of the pulmonary root of the patient #3 in end-systole and end-diastole showing the change in the pulmonary root shape. (**C**) Normalized pulmonary basal ring perimeter, commissural perimeter and sinutubular perimeter lengths during the cardiac cycle. Basal ring perimeter is largest at end-diastole (70–80% of R–R interval). Commissural and sinutubular perimeters are largest at mid systole (30% of R–R interval). Data are limited to the CTA imaging in the 30–80% of the RR interval only.

The muscular pulmonary BR showed large variations in perimeter during the cardiac cycle in all 3 patients. The mean difference between relative maximal and minimal perimeter was 23.5% (SD: 2.7). Pulmonary BR perimeter was shortest at the end-systole and longest at the end-diastole (Fig. 5). The distal vascular part of the pulmonary root (STJ and commissural perimeter) dilated passively with the increase in pulmonary artery pressure. The mean difference between relative maximal and minimal perimeter was 12.0% (SD: 7.0) for STJ perimeter and 14.0% (SD: 4.0) for commissural perimeter (Fig. 5). Similar trends were observed in pulmonary BR area, commissural area and STJ area, showing relative variation of 43.6% (SD: 3.4), 27.0% (SD: 7.0) and 23.0% (SD: 11.0), respectively.

In the same 3 patients, the variations in aortic root perimeters were much smaller. The relative differences for aortic BR perimeter, commissural perimeter and STJ perimeter were 5.6% (SD: 1.6), 3.6% (SD: 1.2) and 3.1% (SD: 2.1), respectively. Relative differences for aortic BR area, commissural area and STJ area were 10.4% (SD: 2.8), 7.0% (SD: 4.0) and 6.0% (SD: 4.0), respectively.

## DISCUSSION

To our knowledge, this is the first *in vivo* investigation analysing pulmonary valve geometry compared to normal aortic valve geometry. Normal diastolic pulmonary valve BR is larger than aortic BR in this *in vivo* study. This was previously shown in 6801 autopsied hearts used for cryopreservation [10]. In the similar BSA range as our study (between 1.5 and 2.4 m^2^), the average cadaveric pulmonary BR diameter was 3.1 mm (SD: 0.2) larger than the aortic BR [10]. In our study, this difference was 5.6 mm (SD: 1.9). The reason for this difference may be that the CTAs were analysed at end-diastole, when right ventricular (and sub-pulmonary infundibular) muscle is actively distended, whereas in cadaveric hearts, the muscle is relaxed but not distended.

Our study has also shown large variations in pulmonary BR, commissural and STJ perimeters during the cardiac cycle. The large diastolic pulmonary BR perimeter and its variability during the cardiac cycle are probably due to its muscular composition and active contraction [14]. Commissural and STJ perimeter changes during the cardiac cycle are opposite to the BR perimeter changes. They apparently dilate passively during systole when pulmonary arterial pressure is rising and decrease in size during diastole when the pressure is falling.

The cyclical changes in aortic perimeters were much smaller. BR perimeter variability was only 5.6% in our study and was even less at the commissural and STJ levels. Hamdan *et al.* [18] have reported a 2.2% BR perimeter change in normal subjects with mean age of 56 years and <1% BR perimeter change in elderly patients with aortic stenosis. The main mechanism of aortic orifice area increase during systole seems to be the shape change due to movement of the aorto-mitral continuity and not increase in perimeter [18].

Anatomical studies have previously shown that the normal aortic and pulmonary valves have approximately equally sized cusps [14]. This was confirmed by our study. Interestingly, pulmonary cusps had lower eH at the end-diastole. This is the result of smaller commissural and STJ diameters and larger BR diameter of the pulmonary root in diastole. Similar observations have been made in a computer simulation study of the aortic valve [19]. The low eH and progressive BR dilatation during diastole may be responsible for the mild or trace pulmonary regurgitation, which has been reported in 40–78% of patients with morphologically normal pulmonary valves [20].

If the pulmonary cusps have similar shape and dimensions as the aortic cusps, changing the pulmonary root to an aortic root-like shape should increase cusp effective height to the values that are normal for the aortic valve. In our series, this would translate to predominant reduction of pulmonary BR and slight increase in pulmonary STJ. This also indicates that for creation of an aortic root-like configuration, as in the Ross operation, annular size reduction to that of a normal aortic annulus should be aimed for. Leaving the pulmonary annulus unrestricted may contribute to autograft regurgitation, even in the absence of secondary dilatation.

Changing the autograft dimensions to improve cusp effective height and maintaining this configuration in the long term is crucial in the Ross procedure in which autograft dilatation leads to regurgitation [6]. Different techniques have been developed to prevent autograft dilatation, such as autologous root inclusion technique [21–23], prosthetic material inclusion technique (Dacron or Ross-PEARS) [24–26] and full root replacement technique with BR and STJ stabilization and wrapping of the autograft with the remnants of aortic wall [27, 28]. Most current techniques already reduce the pulmonary BR to dimensions of a normal aortic BR by implantation in the aortic root, which is either normal *per se* or reduced and stabilized with an annuloplasty.

### Limitations

The study applies only to tricuspid aortic and pulmonary valves. One of the major limitations is the poor pulmonary cusp visibility on CTA, particularly in systole. Another important limitation is the lack of clinical data and comorbidities. The normal function of pulmonary and aortic valves was assumed, based on their appearance on CTA, and was not confirmed by echocardiography.

## CONCLUSION

The pulmonary root has a similar cusp size compared to the aortic root, but a different shape, resulting in a lower pulmonary cusp effective height. The perimeter of the pulmonary BR changes significantly during the cardiac cycle. These findings suggest that stabilizing the autograft to normal aortic, rather than pulmonary, root dimensions should result in normal autograft cusp configuration. CTA could become a tool for personalized planning of the Ross procedure.

## Supplementary Material

ivae206_Supplementary_Data

## Data Availability

The data will be shared on reasonable request.

## Notes

Presented at the 38th EACTS meeting, Lisbon, Portugal, October 2024.

## ABBREVIATIONS

BR: Virtual basal ring of either aortic or pulmonary valve
BSA: Body surface area in m^2^ calculated by Mosteller formula
CTA: Computed tomography angiography
eH: Effective cusp height
NMEC: National Medical Ethics Committee
SD: Standard deviation
STJ: Sinutubular junction of either aortic or pulmonary valve

## References

[1] El-Hamamsy I , Toyoda N, Itagaki S et al Propensity-matched comparison of the Ross procedure and prosthetic aortic valve replacement in adults. J Am Coll Cardiol 2022;79:805–15. 35210036 10.1016/j.jacc.2021.11.057

[2] Yokoyama Y , Kuno T, Toyoda N et al Ross procedure versus mechanical versus bioprosthetic aortic valve replacement: a network meta-analysis. J Am Heart Assoc 2023;12:e8066. 36565200 10.1161/JAHA.122.027715 PMC9973571

[3] Torii R , El-Hamamsy I, Donya M et al Integrated morphologic and functional assessment of the aortic root after different tissue valve root replacement procedures. J Thorac Cardiovasc Surg 2012;143:1422–8. 22361248 10.1016/j.jtcvs.2011.12.034

[4] Abeln KB , Matsushima S, Ehrlich T, Giebels C, Schafers HJ. The Ross procedure versus repair for treatment of a unicuspid aortic valve in adults. Eur J Cardiothorac Surg 2023;64:ezad118. 10.1093/ejcts/ezad118 PMC10320095 36961343

[5] Aicher D , Holz A, Feldner S, Kollner V, Schafers HJ. Quality of life after aortic valve surgery: replacement versus reconstruction. J Thorac Cardiovasc Surg 2011;142:e19–24. 21450311 10.1016/j.jtcvs.2011.02.006

[6] David TE , Omran A, Ivanov J et al Dilation of the pulmonary autograft after the Ross procedure. J Thorac Cardiovasc Surg 2000;119:210–20. 10649195 10.1016/S0022-5223(00)70175-9

[7] Marom G , Weltert LP, Raanani E et al Systematic adjustment of root dimensions to cusp size in aortic valve repair: a computer simulation. Interdiscip Cardiovasc Thorac Surg 2024;38:ivae024. 10.1093/icvts/ivae024 PMC10902611 38402485

[8] Jelenc M , Jelenc B, Poglajen G, Lakic N. Aortic valve leaflet and root dimensions in normal tricuspid aortic valves: a computed tomography study. J Card Surg 2022;37:2350–7. 35526127 10.1111/jocs.16587

[9] Westaby S , Karp RB, Blackstone EH, Bishop SP. Adult human valve dimensions and their surgical significance. Am J Cardiol 1984;53:552–6. 6229998 10.1016/0002-9149(84)90029-8

[10] Kouchoukos NT , Doty DB, Karp RB, Blackstone EH, Hanley FL. Anatomy, Dimensions, and Terminology in Kirklin/Barrat-Boyes Cardiac Surgery. 3rd edn. Philadelphia: Churchill Livingstone, 2003, 36–7.

[11] Santiago MT , Quelroz e Melo J, Ducla-Soares E. Relationships between the dimensions of the human aortic and pulmonary valve leaflets: implications on Ross’ operation. Eur J Cardiothorac Surg 1996;10:599–602. 8875165 10.1016/s1010-7940(96)80372-1

[12] Kelly NF , Platts DG, Burstow DJ. Feasibility of pulmonary valve imaging using three-dimensional transthoracic echocardiography. J Am Soc Echocardiogr 2010;23:1076–80. 20702063 10.1016/j.echo.2010.06.015

[13] Jelenc M , Jelenc B, Habjan S et al Segmental analysis of aortic basal ring dimensions in normal and dilated tricuspid aortic roots. Interdiscip Cardiovasc Thorac Surg 2024;38:ivae029. 10.1093/icvts/ivae029 PMC10927323 38419579

[14] Merrick AF , Yacoub MH, Ho SY, Anderson RH. Anatomy of the muscular subpulmonary infundibulum with regard to the Ross procedure. Ann Thorac Surg 2000;69:556–61. 10735698 10.1016/s0003-4975(99)01300-4

[15] Izawa Y , Mori S, Tretter JT et al Normative aortic valvar measurements in adults using cardiac computed tomography—a potential guide to further sophisticate aortic valve-sparing surgery. Circ J 2021;85:1059–67. 33408304 10.1253/circj.CJ-20-0938

[16] Yang DH , Kim D-H, Handschumacher MD et al In vivo assessment of aortic root geometry in normal controls using 3D analysis of computed tomography. Eur Heart J Cardiovasc Imaging 2017;18:780–6. 27461206 10.1093/ehjci/jew146 PMC6279090

[17] Jelenc M , Jelenc B, Habjan S et al Aortic valve cusp size and shape in dilated trileaflet aortic roots. J Thorac Cardiovasc Surg 2024;S0022-5223(24)00621-4. 10.1016/j.jtcvs.2024.07.021 39032628

[18] Hamdan A , Guetta V, Konen E et al Deformation dynamics and mechanical properties of the aortic annulus by 4-dimensional computed tomography: insights into the functional anatomy of the aortic valve complex and implications for transcatheter aortic valve therapy. J Am Coll Cardiol 2012;59:119–27. 22222074 10.1016/j.jacc.2011.09.045

[19] Marom G , Haj-Ali R, Rosenfeld M, Schafers HJ, Raanani E. Aortic root numeric model: annulus diameter prediction of effective height and coaptation in post-aortic valve repair. J Thorac Cardiovasc Surg 2013;145:406–11 e1. 22365065 10.1016/j.jtcvs.2012.01.080

[20] Lancellotti P , Pibarot P, Chambers J et al; Scientific Document Committee of the European Association of Cardiovascular Imaging. Multi-modality imaging assessment of native valvular regurgitation: an EACVI and ESC council of valvular heart disease position paper. Eur Heart J Cardiovasc Imaging 2022;23:e171–232. 35292799 10.1093/ehjci/jeab253

[21] Ross DN. Homograft replacement of the aortic valve technique. Br J Surg 1967;54:165–8. 6020983 10.1002/bjs.1800540303

[22] Mazine A , El-Hamamsy I, Verma S et al Ross procedure in adults for cardiologists and cardiac surgeons: JACC state-of-the-art review. J Am Coll Cardiol 2018;72:2761–77. 30497563 10.1016/j.jacc.2018.08.2200

[23] Skillington PD , Mokhles MM, Takkenberg JJ et al The Ross procedure using autologous support of the pulmonary autograft: techniques and late results. J Thorac Cardiovasc Surg 2015;149:S46–52. 25439787 10.1016/j.jtcvs.2014.08.068

[24] Slater M , Shen I, Welke K, Komanapalli C, Ungerleider R. Modification to the Ross procedure to prevent autograft dilatation. Semin Thorac Cardiovasc Surg Pediatr Card Surg Annu 2005;181–4. 15818376 10.1053/j.pcsu.2005.01.022

[25] Jahanyar J , Munoz DE, Tamer S, El Khoury G, de Kerchove L. The Ross inclusion Dacron graft. Ann Cardiothorac Surg 2021;10:549–51. 34422574 10.21037/acs-2020-rp-17 PMC8339629

[26] Carrel T , Kadner A. Long-term clinical and imaging follow-up after reinforced pulmonary autograft Ross procedure. Semin Thorac Cardiovasc Surg Pediatr Card Surg Annu 2016;19:59–62. 27060045 10.1053/j.pcsu.2015.11.005

[27] Mazine A , El-Hamamsy I. Tailoring the Ross procedure for patients with aortic regurgitation. JTCVS Tech 2021;10:383–9. 34977760 10.1016/j.xjtc.2021.06.008 PMC8690315

[28] Matsushima S , Abeln KB, Karliova I, Zacek P, Schafers HJ. Suture annuloplasty and simplified root wrapping in the full root Ross operation. Ann Thorac Surg 2019;107:e361–63. 30582923 10.1016/j.athoracsur.2018.11.048

