# Supplementary material for: Comparison of pulmonary and aortic root and cusp dimensions in normal adults using computed tomography: potential implications for Ross procedure planning
Source: Interdiscip Cardiovasc Thorac Surg. 2024 Dec 9;39(6):ivae206. doi: 10.1093/icvts/ivae206 (PMC11665635; doi:10.1093/icvts/ivae206)
Supplement: ivae206_Supplementary_Data [file ivae206_supplementary_data.docx]

**Supplementary table S1.** Correlations between pulmonary and aortic root and cusp measurements stratified by sex

|  | All (n=50) | Male (n=27) | Female (n=23) |
| --- | --- | --- | --- |
|  | Pearson’s r (P-value) | Pearson’s r (P-value) | Pearson’s r (P-value) |
| Basal ring diameter | 0.790 (<0.001) | 0.767 (<0.001) | 0.575 (0.004) |
| Sinutubular diameter | 0.515 (<0.001) | 0.368 (0.059) | 0.563 (0.005) |
| Commissural height | 0.758 (<0.001) | 0.693 (<0.001) | 0.460 (0.027) |
| Geometric cusp height | 0.727 (<0.001) | 0.477 (0.012) | 0.575 (0.004) |
| Effective cusp height | 0.294 (0.038) | 0.268 (0.177) | 0.367 (0.085) |
| Cusp insertion length | 0.880 (<0.001) | 0.806 (<0.001) | 0.732 (<0.001) |
| Free margin length | 0.789 (<0.001) | 0.611 (<0.001) | 0.765 (<0.001) |
| Pulmonary BR and BSA | 0.674 (<0.001) | 0.465 (0.015) | 0.507 (0.014) |
| Aortic BR and BSA | 0.735 (<0.001) | 0.441 (0.021) | 0.505 (0.014) |

BR – basal ring, BSA – body surface area


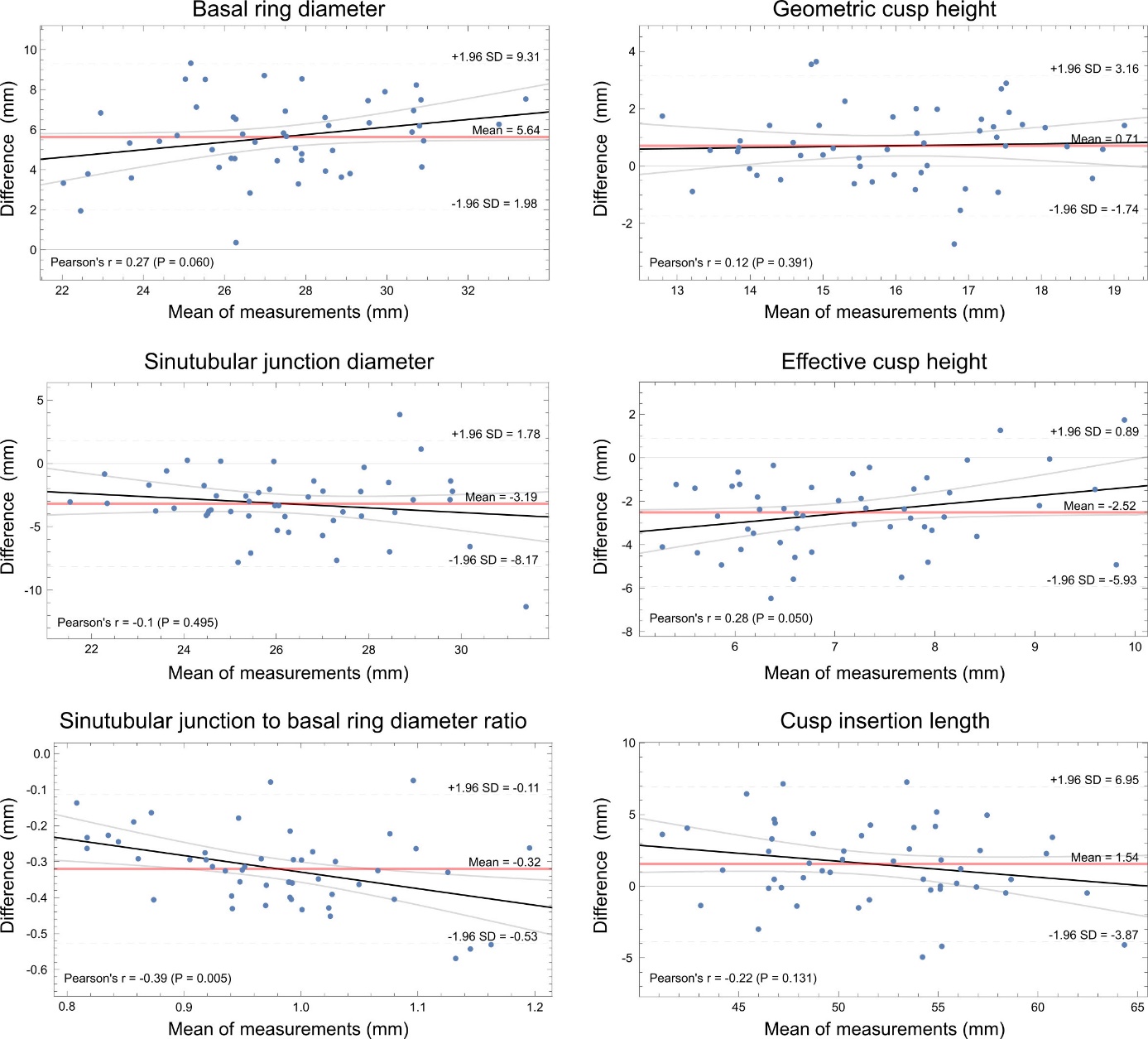


**Supplementary Figure S1**. Bland-Altman plots of individual patient differences between pulmonary and aortic measurements.
